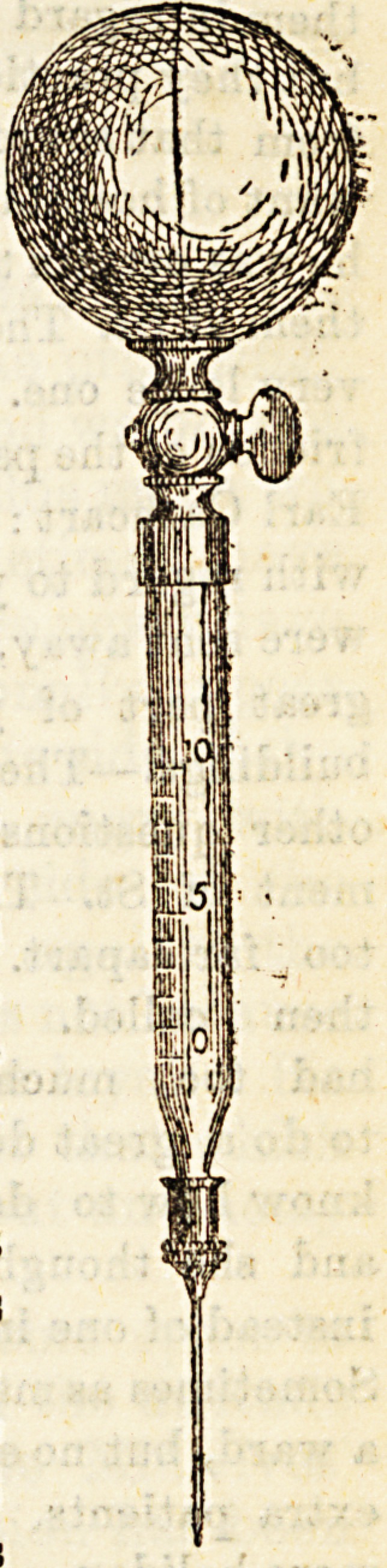# New Drugs, Appliances, and Things Medical

**Published:** 1891-02-14

**Authors:** 


### NEW DRUGS, APPLIANCES, AND THINGS MEDICAL.

[All preparations, appliances, novelties, etc., of which a notice is desired, should be sent for The Editor, to care of The Manager, 140, Strand, London, W.C.]

### DR. KOCH.

Messrs. Hockin, Wilson, and Co., druggists, New Inn Yard, have courteously forwarded copies of their photographs of Professor Koch. We think they have succeeded admirably in portraying the Doctor's appearance, and we are informed that on receipt of three stamps they will have pleasure in sending a copy to any medical man who applies for it. By the same post we received a specimen of the hypodermic syringe used by the Professor in treating his patients. This is made by Ferris and Co., Bristol. As will be seen by the woodcut, the syringe consists of three parts—an india rubber ball with German-silver tap attached, a graduated glass cylinder tapered at one end, and an ordinary hypodermic needle. The larger end of the cylinder fits air-tight into the mouth of the tap, and the needle fits over the smaller end of the cylinder. The cylinder with needle attached is filled with fluid for injection, preferably by means of a pipette, and fixed firmly to the ball, the tap being closed. The needle is then introduced beneath the skin, the tap opened, and the injection made by pressing the india-rubber ball. The graduated portion of the cylinder (0—10) holds 1 gramme or 1 cc. of liquid, each division being equal to 0·1 cc. Messrs. Ferris and Co. state that this pattern syringe has been submitted to and approved by Dr. Koch. They also supply graduated measures and pipettes for making the dilutions and filling the syringe. The lymph or fluid is usually employed in three dilutions—1 in 10, 1 in 100, and 1 in 1000, made with half per cent. carbolic acid solution. Therefore, one syringeful (0—10) of the 1 in 10 equals 0·1 cc. of the original fluid; one syringeful (0—10) of the 1 in 100 equals 0·01 cc. of the original fluid; one syringeful (0—10) of the 1 in 1,000 equals 0·001 cc. of the original fluid. The instrument is well made and very handy and portable.

### SANITAS PREPARATIONS.

The Sanitas Antiseptic Toilet Soap is one of the most useful preparations in the market. We have tried it in obstinate cases of eczema of the palm, and have found it has succeeded in keeping the skin healthy where everything else has failed. In those cases where the eczema is severe an application of carbolic and glycerine in the proportion of 3 to 2 is very helpful, and if combined with the continuous use of Sanitas Soap will keep the patient free from any return. One great advantage that Sanitas has is its pleasant smell, whilst its effectiveness is now established beyond all question. Amongst other preparations we may mention Sanitas Oil, Sanitas Jelly, Sanitas Powder and Fluid; also a disinfecting furniture cream, which is much pleasanter to use than the ordinary polish. One of the preparations we have not had the courage to tackle. It is the Peroxide of Hydrogen, which is described as imparting "the desired golden shade to the human hair." Dr. Kingzett apparently makes this suggestion quite seriously, and those who favour golden hair might possibly use it to much greater advantage than many of the dyes so much in vogue. Sanitas has been so long before the public and is so well known that it is almost superfluous to add that it is now widely recognised as one of the most popular and pleasant disinfectants.

## Figures and Tables

**Figure f1:**